# Primary human ovarian interstitial cells contribute to murine follicle growth through follicle-interstitial paracrine crosstalk

**DOI:** 10.21203/rs.3.rs-7593401/v1

**Published:** 2025-09-24

**Authors:** Elizabeth L. Tsui, Diane C. Saunders, Lina Lu, Tara Kennedy, Sunkyung Lee, Szymon K. Filip, Peter A. Faull, Neil Hunter, Erin E. Rowell, Ruli Gao, Monica M. Laronda

**Affiliations:** a Stanley Manne Children’s Research Institute, Ann & Robert H. Lurie Children’s Hospital of Chicago, Chicago, USA 60611; b Department of Pediatrics, Feinberg School of Medicine, Northwestern University, Chicago, IL, USA, 60611; c Department of Biochemistry and Molecular Genetics, Northwestern University, Chicago, IL, USA, 60611; d Department of Surgery, Feinberg School of Medicine, Northwestern University, Chicago, IL, USA, 60611; e Department of Microbiology and Molecular Genetics, University of California, Davis, Davis, California, USA, 95616; f Howard Hughes Medical Institute, University of California, Davis, Davis, California, USA, 95616; g Northwestern University Proteomics Core, Proteomics Center of Excellence, Northwestern University, Chicago, IL, USA, 60611; h Department of Obstetrics and Gynecology, Feinberg School of Medicine, Northwestern University, Chicago, IL, USA, 60611

## Abstract

Paracrine signaling is critical for ovarian development and maintenance of non-growing, gonadotropin-independent follicles. During puberty the human ovary develops defined compartments as gonadotropin signaling triggers follicle activation and maturation. Adjacent follicles utilize paracrine signaling but also benefit from nearby interstitial cell populations. Here, we characterized human ovarian interstitial cells and their effects on follicle growth through a cross-species culture assay. Both prepubertal and postpubertal primary ovarian interstitial populations improved follicle growth, and postpubertal cells conferred a significant increase in follicle estradiol production but not maturation rates. In one cohort, interstitial cell-conditioned media was not sufficient for the growth advantage observed with co-culture. To investigate how follicle-interstitial cell crosstalk changes during puberty, single-cell RNA-sequencing was performed on 11 ovarian tissue cryopreservation samples (participants 0.34–22.8 years). Results confirmed the presence of heterogenous interstitial cell populations previously identified in the adult ovary, including abundant theca/stromal cells expressing genes enriched for ECM-related processes. Finally, over 100 proteins were identified in co-culture media using bottom-up proteomics representing the human ovarian interstitial secretome. This study uniquely characterized human ovarian interstitial cells across the pubertal transition and advances our understanding of the human pediatric and adolescent ovarian follicular microenvironment.

## INTRODUCTION

Ovarian health plays a significant role in optimal function of cardiovascular, bone, and metabolic systems, leading to lifelong implications for individuals with compromised fertility due to genetic, environmental, or iatrogenic factors such as those undergoing gonadotoxic chemotherapy for cancer treatment. Development of and improved outcomes for fertility preservation and hormone restoration technologies rely on understanding changes in the ovarian microenvironment, particularly during puberty. This developmental transition includes maturation of the hypothalamic-pituitary-gonadal axis, establishment of ovarian subanatomy, and the cyclical activation of cohorts of quiescent oocytes from the finite ovarian reserve established at birth to produce mature, fertilizable eggs^1–8^.

Mechanisms of human oocyte activation and development, collectively referred to as folliculogenesis, remain incompletely understood. Lineage tracing studies in murine models have demonstrated that a subset of granulosa and theca cells, steroidogenic cells surrounding the oocytes that define the functional unit of a follicle, derive from perivascular and additional interstitial cell populations^9,10^. Furthermore, paracrine signaling from a theca- and macrophage-enriched murine interstitial population can support the growth and survival of isolated follicles *in vitro*^11^. Subsequent studies have reported improved follicle growth in response to other feeder populations including murine embryonic fibroblasts, adipose-derived stem cells, and peritoneum mesothelial stem cells^12–17^. In addition, blood vessel recruitment and extracellular matrix (ECM) remodeling is necessary to accommodate physical expansion of a growing follicle and, eventually, ovulation^18–20^.

Mounting evidence suggests that interstitial cells and their secreted products are important for optimal folliculogenesis in the human ovary. Amorim and colleagues recently reported that isolated preantral follicles cultured with ovarian stromal cells from postmenopausal donors resulted in increased growth, survival, and estradiol production^21^. Furthermore, maturation outcomes of oocytes cultured *in vitro* are most successful when small follicles are grown *in situ*, relative to methods in which the ECM or cell-cell communication is disrupted through enzymatic and/or mechanical isolation techniques^22–24^. However, limited data exists regarding the interstitial cell composition of the human ovary and its associated secretome^15,16,25–28^, which are likely to drive the growth and maturation outcomes observed. Previous work utilized human ovarian tissue collected during ovarian tissue cryopreservation (OTC) to characterize the human ovarian subanatomy and microenvironment^8^. These analyses revealed the dynamic gross histological changes that occur in the ovary during the pubertal transition. Additionally, it was shown that rates of *in vitro* maturation and euploidy were lower for oocytes isolated from individuals less than 21 years old^29^. Here, we add to the growing body of knowledge that recognizes the pubertal transition and adolescence as a critical window of the reproductive lifespan by characterizing the cell types and secretome of interstitial cells from human ovaries. This study demonstrates a beneficial growth effect of primary human ovarian interstitial cells on murine follicle growth and incorporates single-cell RNA-sequencing (scRNA-seq) of pediatric and adolescent ovarian tissue to define stromal cell subpopulations resembling those found in adult ovaries. Using both transcriptomic and proteomic analyses, we identify conserved proteins whose secretion impacts follicle growth.

## RESULTS

### Primary human ovarian interstitial cells improved growth of isolated and encapsulated murine secondary follicles

**Figure 1.**
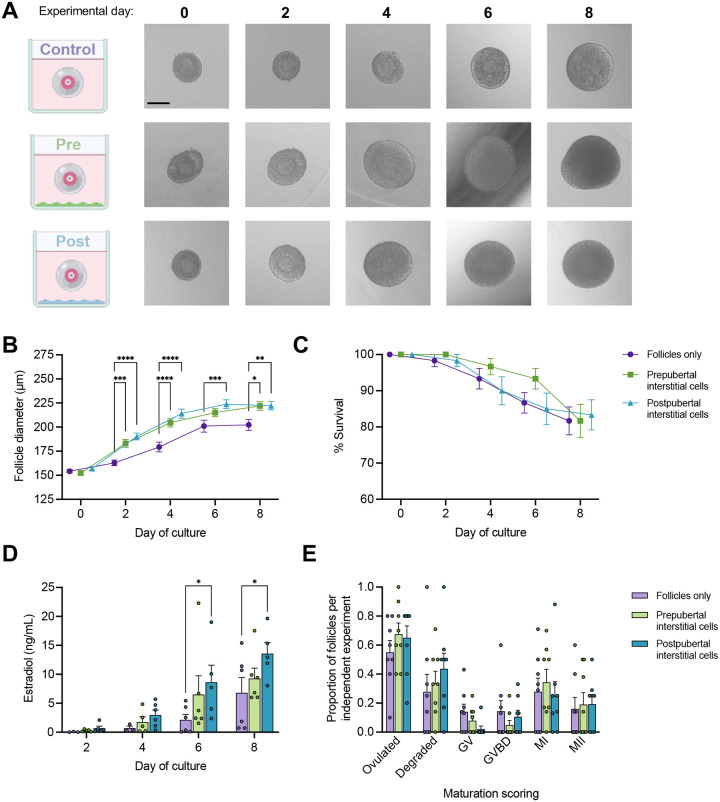
Co-culture of isolated murine ovarian follicles with human ovarian interstitial cells. (A) Schematic of control and co-culture conditions and representative brightfield microscopy images of all conditions at day 0, 2, 4, 6, and 8 days of culture. Scale bar, 100 μm. (B-C) Follicle diameter (B) and survival (C) over 8 days of culture, mean ± SEM. (D) Estradiol concentration measured in spent media from all conditions, mean + SEM. (E) Oocyte maturation outcomes of cultured follicles, mean + SEM. GV, germinal vesicle; GVBD, germinal vesicle breakdown; MI, metaphase I oocyte; MII, metaphase II oocyte. (B-E) Mixed-effects analysis or 2-way ANOVA with Tukey’s multiple comparisons test: ****, P <0.0001; ***, P <0.001; **, P < 0.01; *, P < 0.05.

Murine ovarian secondary follicles (starting diameter 150.1–154.3 μm) were cultured in an established 3-dimensional (3D) system with alginate encapsulation alone (control) or in the presence of a feeder layer of human ovarian interstitial cells to assay the effect of paracrine signaling on follicle growth dynamics, oocyte maturation outcomes, and estradiol production. Human ovarian interstitial cells derived from prepubertal (‘Pre’) and postpubertal (‘Post’) participants were additional subgroups to evaluate for functional differences across the pubertal transition. (Figure 1A and **Supplementary Table 1**). Follicles co-cultured with interstitial cells grew to larger diameters than follicles grown in alginate alone beginning at day 2 of culture (Figure 1B–1C). By day 8, control follicle diameters averaged 202.5 ± 6.5 μm compared to 225.6 ± 5.1 μm and 227.7 ± 5.3 μm from prepubertal and postpubertal co-culture groups, respectively. There was an average survival above 80% in each group (Figure 1C). Increased growth of follicles co-cultured with interstitial cells from postpubertal ovaries were accompanied by a significant increase in estradiol production over control at days 6 and 8 of culture (Figure 1D). To determine if the larger follicles in our co-culture groups produced more eggs upon a human chorionic gonadotropin (hCG) trigger, follicles were removed from alginate, matured *in vitro,* and scored by morphology. No statistically significant difference in the number of ovulated oocytes, degraded, germinal vesicle (GV), germinal vesicle breakdown (GVBD), metaphase I (MI) oocytes or metaphase II (MII) eggs was observed between groups (Figure 1E, **Supplementary Figure 1A**). While interstitial cells were not isolated from ovaries of individuals exposed to alkylating agent therapies that would put them at “significant” or “high” increased risk of gonadal dysfunction^30^, a subset of participants had some previous exposure to alkylating chemotherapies (**Supplementary Table 1**). When follicle growth outcomes were stratified by previous exposure to alkylating chemotherapy, no statistically detectable effect of previous chemotherapy exposure on follicle growth was found (**Supplementary Figure 1B**).

### Interstitial cell conditioned media was not sufficient to improve follicle growth in 8-day culture

**Figure 2.**
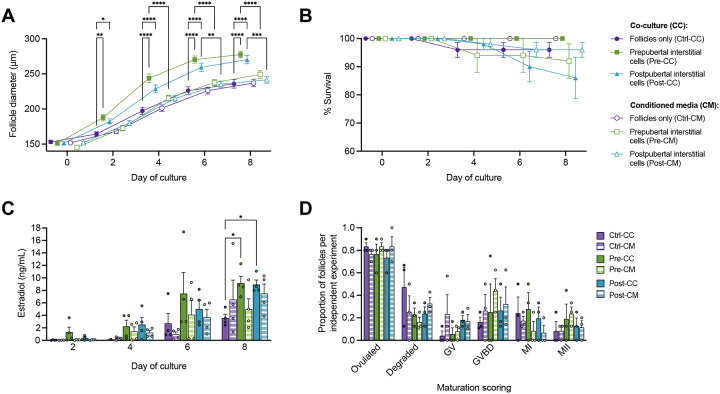
8-day co-culture and conditioned media experiments of isolated murine ovarian follicles with human ovarian interstitial cells. (A-B) Follicle diameter (A) and survival (B) over 8 days of culture, mean ± SEM. (C) Estradiol concentration measured in spent media from all conditions, mean + SEM. (D) Oocyte maturation outcomes of cultured follicles, mean + SEM. GV, germinal vesicle; GVBD, germinal vesicle breakdown; MI, metaphase I oocyte; MII, metaphase II oocyte. (A-D) Mixed-effects analysis or 2-way ANOVA with Tukey’s multiple comparisons test: ****, P <0.0001; ***, P <0.001; **, P < 0.01; *, P < 0.05.

To determine whether human interstitial cells required the presence of follicles to secrete paracrine factors that promote follicle growth, the experimental design was expanded to include conditioned media (CM) in parallel with the co-culture (CC) experiments as previously performed (**Supplementary Figure 2**). Survival rates of follicles were 86% to 100% across conditions after 8 days in culture (Figure 2A and 2B). As before, mean follicle diameter did not differ significantly between Pre-CC and Post-CC groups; however, follicles in both CC groups grew to larger diameters than controls as well to their corresponding CM conditions (Pre-CC vs Pre-CM, P < 0.0001; Post-CC vs Post-CM, P = 0.0006) (Figure 2A). Follicles grown in media conditioned by either prepubertal (Pre-CM) or postpubertal (Post-CM) interstitial cells had no growth advantage when compared to control follicles (Figure 2A). Estradiol production increased in all groups over time (P < 0.0001), though only follicles grown in co-culture produced significantly more estradiol compared to controls at day 8 (Figure 2C). Total ovulation events and maturation stage distributions were similar across all conditions (Figure 2D). MII to total follicle ratios were 0.083 ± 0.083, 0.190 ± 0.132, and 0.125 ± 0.072 for control, Pre-CC, and Post-CC follicles, respectively (Figure 2D). MII to total follicle ratios were 0.083 ± 0.042, 0.236 ± 0.061, and 0.114 ± 0.059 for control, Pre-CM, and Post-CM follicles, respectively (Figure 2D).

**Figure 3.**
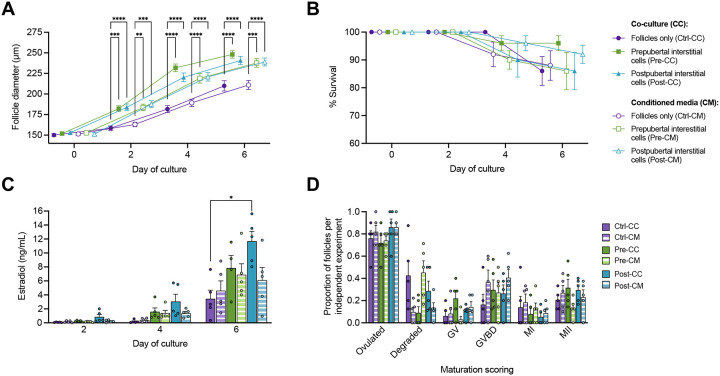
6-day co-culture and conditioned media experiments of isolated murine ovarian follicles with human ovarian interstitial cells. (A-B) Follicle diameter (A) and survival (B) over 6 days of culture, mean ± SEM. (C) Estradiol concentration measured in spent media from all conditions, mean + SEM. (D) Oocyte maturation outcomes of cultured follicles, mean + SEM. GV, germinal vesicle; GVBD, germinal vesicle breakdown; MI, metaphase I oocyte; MII, metaphase II oocyte. (A-D) Mixed-effects analysis or 2-way ANOVA with Tukey’s multiple comparisons test: ****, P <0.0001; ***, P <0.001; **, P < 0.01; *, P < 0.05.

Since follicle growth rates peaked between days 2 and 4 in culture and follicles in the Pre- and Post-CC conditions had already reached a diameter appropriate for maturation by day 6, co-culture and conditioned media experiments were replicated using a different cohort of participant-derived tissue on a shorter timeframe (maturing follicles at day 6 instead of day 8) to evaluate whether maturation outcomes were affected by more rapid growth. Follicles in all conditions grew over time in culture (P < 0.0001), with average survival above 86% in all culture conditions (Figure 3A and 3B). Consistent with previous experiments, follicles grown in co-culture demonstrated a growth advantage when compared to control follicles, with no differences in growth between Pre-CC and Post-CC groups (Figure 3A). In contrast to day 8 experiments, however, follicles grown in conditioned media also had larger diameters than controls from day 2 to day 6 of culture, with no statistically significant difference between CC and CM conditions for either Pre or Post groups at any experimental day (Figure 3A). Estradiol levels increased throughout culture, though only follicles co-cultured with postpubertal interstitial cell populations produced significantly more estradiol than control follicles at day 6 (Figure 3C). Overall ovulation rates were similar among all groups (Figure 3C). No statistically significant difference in maturation outcomes between control follicles and CC or CM follicles was observed (Figure 3D).

### scRNA-seq of human ovarian tissue reveals heterogenous interstitial cell population primarily composed of theca/stromal subsets

**Figure 4.**
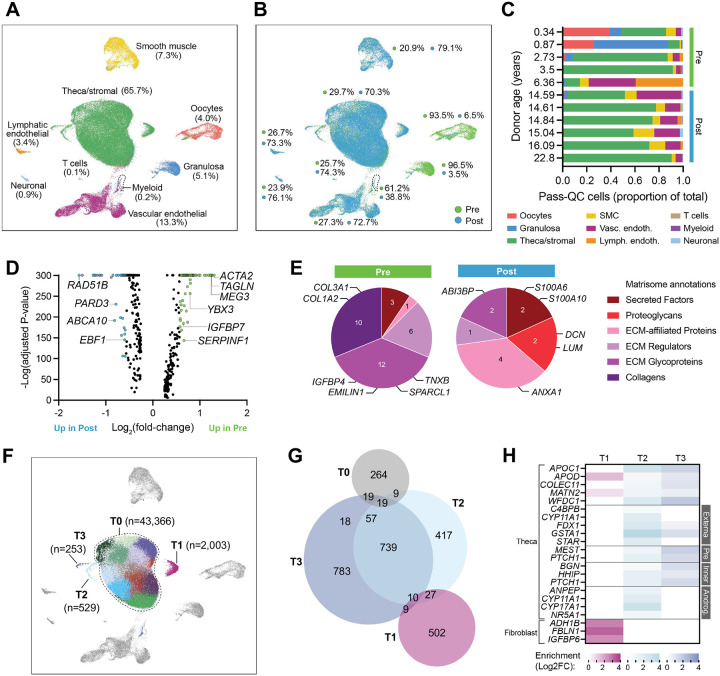
scRNA-seq of human ovarian tissue from pediatric and adolescent individuals. (A-B) Uniform Manifold Approximation Projection (UMAP) of all pass-QC cells colored by cell annotation (A) or puberty status (B; green, prepubertal and blue, post-pubertal). (C) Proportions of annotated cell types per participant ordered by participant age. (D) Volcano plot of theca/stromal cell genes differentially expressed between prepubertal (Pre) and postpubertal (Post) samples; colored symbols denote ≥1.5 fold-change with selected genes labeled. (F) Matrisome-annotated^50^ significantly enriched genes in Pre and Post theca/stromal cells. The top 6 genes per group (by magnitude fold-change) are listed according to their annotations. (F) UMAP colored by original unsupervised clustering, with four main theca/stromal subpopulations labeled (T0 encompasses 10 clusters). (G) Venn diagram showing number of genes significantly enriched per theca/stromal subpopulation, including genes shared between subpopulations. Created with DeepVenn^107^. (H) Heat map showing enrichment of theca and fibroblast markers in subpopulations T1–T3; none of the genes listed were significantly enriched in T0. Theca markers are grouped based on reported specificity for theca externa^32^, pre-theca cells (‘Pre’)^32^, inner theca cells^61^, and androgenic (‘Androg.’) theca cells^61^.

Key to understanding how human interstitial populations influence folliculogenesis is identification of different cell types and states; however, beyond identification of endothelial, smooth muscle, and immune cells based on expression of canonical genes conserved across tissues, human ovarian interstitial cell populations are not yet well described. To address this gap, we generated a scRNA-seq dataset of ovarian tissue biopsies representing cortex and medulla compartments from 11 individual participants (0.34 to 22.80 years of age, n=5 prepubertal and n=6 postpubertal; **Supplementary Table 1**). Our dataset included 85,999 total cells that passed quality control filters, 29,941 cells from prepubertal samples and 56,058 from postpubertal (**Supplementary Table 2A** and **Supplementary Figure 3A**). Unbiased clustering and visualization by uniform manifold approximation and projection (UMAP) revealed cell clusters that represented 9 major cell types: oocytes, granulosa cells, theca/stromal cells, smooth muscle cells (SMCs), vascular endothelial cells, lymphatic endothelial cells, T cells, myeloid cells, and neuronal cells (Figure 4A). Prepubertal samples were enriched in ovarian follicle cell types (oocyte and granulosa cells) and myeloid cells when compared to postpubertal samples, which were enriched in theca/stromal cells, SMCs, neuronal cells, T cells, and endothelial and lymphatic endothelial cells (Figure 4B, 4C, and **Supplementary Figure 3B**).

Cells within each cluster abundantly expressed marker genes for these cell types previously reported in the adult human ovary or associated with cell function in other organisms: *PDCD5* and *ZP3* in oocytes^31–33^, *PTGDS*, *NPY*, and *GATM* in granulosa cells^34–37^, *DCN* and *OGN* in theca/stromal cells^32,33,35,38,39^, *CRIP1*, *PRKG1*, and *RERGL* in SMCs^32,33,37,40^, *VWF* and *CCL14* in vascular endothelial cells^32,33,35^, and *CCL21* and *MMRN1* in lymphatic endothelial cells^41^ (**Supplementary Figure 3C**). Small populations (each less than 1% of all cells) were identified as T cells (*IL7R*)^33,35,38^, myeloid cells (*AIF1*, *C1QA*)^33^, and neuronal cells (*NRXN1*)^37^. Enriched genes per cluster can be found in **Supplementary Table 2B**. A small proportion of oocytes were captured in this dataset, chiefly from prepubertal participants below the age of 2 years, which is consistent with the high follicle density observed at this age^8^. Gene markers highly expressed by these oocytes included *ZFAND2A*, previously identified as upregulated in GV oocytes^42^, *TUBB8*, a GWAS variant^43^, *FIGLA*, upregulated in quiescent follicles^44^, and *UCHL1*, an oocyte-specific transcript identified in many species^45–49^ and implicated in oocyte quiescence in the mouse^47^.

Since ovarian interstitial cells derived from prepubertal and postpubertal participants conferred similar advantages to growth of co-cultured murine follicles, we compared transcriptomic signatures of the largest interstitial population, theca/stromal cells, between the two groups (**Supplementary Table 2C**). 382 genes were significantly differentially regulated, 196 more abundant in prepubertal and 186 more abundant in postpubertal samples. About a quarter of these genes (89 or 23%) were 1.5-fold more abundant (Figure 4D). Upregulated genes in prepubertal samples were highly enriched for epithelial to mesenchymal transition, vasculature development, and response to growth factor pathways (**Supplementary Figure 3D**). Interestingly, 16% of genes enriched in prepubertal theca/stromal cells encoded for ECM-related proteins^50^ compared to only 6% of genes enriched in postpubertal theca/stromal cells (Figure 4E). Among the prepubertal-enriched ECM proteins was ovarian-dominant collagen VI (*COL6A1*, *COL6A2*, *COL6A3*) as well as laminin-associated basement membrane protein nidogen-1 (*NID1*) and adhesion molecule tenascin-X (*TNXB*), all of which have been previously reported to be enriched in the ovarian proteome at the prepubertal stage and are highlighted for their contribution to maintaining follicle dormancy^51^. Response to growth factor genes *KLF10* and *SOCS3* are associated with lipid^52^ and leptin^53^ signaling respectively, while *NR4A1* is a known regulator of granulosa cell differentiation and preserved ovarian function^54,55^. In contrast, genes more abundant in postpubertal samples were highly enriched for eukaryotic translation elongation, reactive oxygen species signaling, and P2X7 receptor signaling (**Supplementary Figure 3E**). Translation-related genes included *CYB5A*, previously detected in antral and pre-ovulatory follicles^56^, and *PRL24*, which has been linked to oocyte developmental competence^57^. Also consistent with proteomics data from Ouni and colleagues^51^ was the enrichment of *ANXA1*, *S100A6*, and *CSTB* in postpubertal theca/stromal cells. *ANXA1* and *S100A6* are thought to regulate follicle activation by modulating phosphorylation modulation while *CSTB* is linked to the inhibition of reactive oxygen species, a signaling event affecting ovulation^58,59^.

### Investigation of theca/stromal cell subsets revealed fibroblast-like and theca-specific signatures

To date there has not been extensive exploration of ovarian stromal cell subtypes, with most studies noting the heterogenous composition and difficulty resolving theca cells from other stromal cell types. A few datasets have described subsets driven by differential gene expression; one study of metastatic high-grade serous ovarian carcinoma, which included a non-diseased sample for comparison, delineated five fibroblast subtypes^60^, while single-cell studies of healthy ovarian tissue have noted stromal subtypes^40^ or combined theca and stroma subtypes by subclustering^33^. Consistent with these observations, mesenchymal and/or stromal cell markers *LUM*, *C7*, *OGN*, and *PDGFRA* were widely expressed by our theca/stromal-annotated cells (**Supplementary Figure 4A**). However, we noticed that original clustering included three small subpopulations within our theca/stromal annotation, so we revisited all theca/stromal-annotated cells from the lens of 4 major groups (T0–T4, Figure 4F–G). The largest and central cluster, T0 (43,366 cells; 74.8% of total theca/stromal cells), was the most heterogenous, with enrichment for ovarian stromal marker genes *NR4A1*, *EGR1*, and *PEG3*, as well as some stress markers (*JUNB*, *FOS*) that may indicate atretic follicular cells^35,39^ (**Supplementary Table 2D**). This group was also enriched for a few perivascular markers (*MYH11*, *TAGLN*), fibroblast activation marker *VIM*, and notably for ECM regulators *TIMP2*, *HTRA1*, and *ADAMTS1* (**Supplementary Table 2D**). It most closely resembled the Fibro_4 fibroblast^60^, ST3 stromal^40^, and Theca & stromal 3^33^ subsets previously identified, driven by enrichment of *EGR1*, *FOS*, *JUN*, and *IER2* (**Supplementary Figure 4B-D**). Genes upregulated in T0 were enriched for FGF, CRH, EGF, and VEGFA signaling pathways, which closely aligned to the “growth factor” descriptor of the Fibro_4 subpopulation^60^ (**Supplementary Figure 4B, 5A**).

Cluster T1 (2,003 cells; 3.5% of total theca/stromal cells) was enriched in genes highly expressed by fibroblast populations including *ABCA6*, *ABCA8*, *ADH1B*, *CCDC80*, *DCN*, *FBLN1, IGFBP6,* and *PRRX1* (Figure 4H, **Supplementary Table 2D**). The T1 subcluster appeared similar to the previously defined Fibro_3^60^ and Theca & stromal 4^33^ populations, with notable overlap in high enrichment of *IGFBP6* and *MGP* and abundance of ECM-related genes, particularly glycoproteins (**Supplementary Table 2D**, **Supplementary Figure 5**). Clusters T2 (529 cells; 0.9% of total theca/stromal cells) and T3 (253 cells, 0.6% of total theca/stromal cells), closely resembling the Theca & stromal 2^33^ population, were enriched in theca marker genes *APOC1*, *HHIP*, *MATN2*, *PTCH1,* and *WFDC1* (Figure 4H, **Supplemental Figure 4D, Supplementary Table 2D**). Androgenic genes *ANPEP*, *CYP11A1*, *CYP17A1*, *NR5A1*, and *STAR* were uniquely enriched in cluster T2^61^, as was *C4BPB*, previously described as a theca externa marker^35^ (Figure 4H, **Supplementary Table 2D**). Cells in T3 were highly enriched for theca markers *BGN* and *COLEC11*, as well as several mitosis genes (e.g., *CCNB1*, *CENPE*, *NUSAP1*, *RAN*, *TOP2A*) and theca progenitor marker *MEST*^35^ (Figure 4H, **Supplementary Table 2D**). Additionally, 12 of the 19 genes that demonstrated high enrichment (>4 fold-change) correspond to proteins that are either known to be or predicted to be secreted^62^. Together, these data indicate that the theca/stromal cell population is quite heterogenous even in the prepubertal ovary, with multiple sub-populations that have relatively small differences in gene expression.

### Computational analysis of ligand-receptor pairs predicted paracrine interactions between interstitial cell types

**Figure 5.**
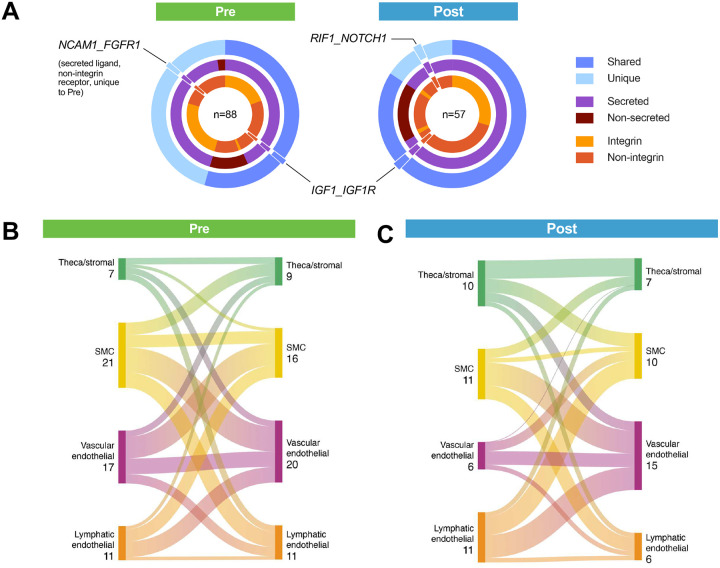
Putative cell-cell communication networks using transcriptome ligand-receptor analyses. (A) Ligand-receptor pairs identified in Pre and Post ovarian tissue datasets. Each concentric circle shows one of the following: shared or unique between datasets, ligands classified as secreted or non-secreted, receptors classified as integrins or non-integrins. Total predicted pairs listed as n. (B-C) Sankey plots of unique ligand-receptor pairs detected in Pre (B) and Post (C) interstitial cells. Low-abundance cell types (T cells, myeloid cells, neuronal cells) were excluded. Created using SankeyMATIC.

Cell populations within a tissue exhibit cell-cell communication to carry out intricate organ-level functions. To explore the cell-cell communication networks predicted within our dataset, we analyzed putative secreted ligand-receptor pairs among theca/stromal cells, smooth muscle cells, vascular endothelial cells, and lymphatic endothelial cells using CellPhoneDB^63^. Oocytes and granulosa cells captured in the dataset were predominantly from two prepubertal samples, so they were excluded from the analysis. In this cohort of participants, prepubertal samples had more statistically significant ligand-receptor pairs (88) when compared to postpubertal samples (57), with most pairs including a secreted ligand (Figure 5A). The most common source of putative secreted ligands in prepubertal samples was smooth muscle cells, accounting for 37% of total interactions, followed closely by vascular endothelial cells (30%) (Figure 5B). Interstitial cell-produced ligands corresponded most to receptors on vascular endothelial cells (36%) and smooth muscle cells (28%).

Secreted ligands in postpubertal samples were more evenly distributed among interstitial cell types (smooth muscle cells and lymphatic endothelial cells represented 29% each, with theca/stromal cells at 26%). These ligands are known to bind to receptors expressed mostly by vascular endothelial cells (39%) (Figure 5C). Interestingly, the most apparent difference in postpubertal compared to prepubertal samples was a greater abundance in autocrine signaling within theca/stromal cells where 57% of signaling mediated through theca/stromal cell-expressed receptors was derived from theca/stromal cell-expressed ligands. Overall, both prepubertal and postpubertal theca/stromal cells expressed receptors predicted to transduce secreted signals from all other interstitial cell types identified within the ovary and are thus likely critical mediators of organ-wide growth and homeostasis.

### Interstitial cells in culture secreted numerous proteins related to ECM modulation and hormone-sensitive signaling

**Figure 6.**
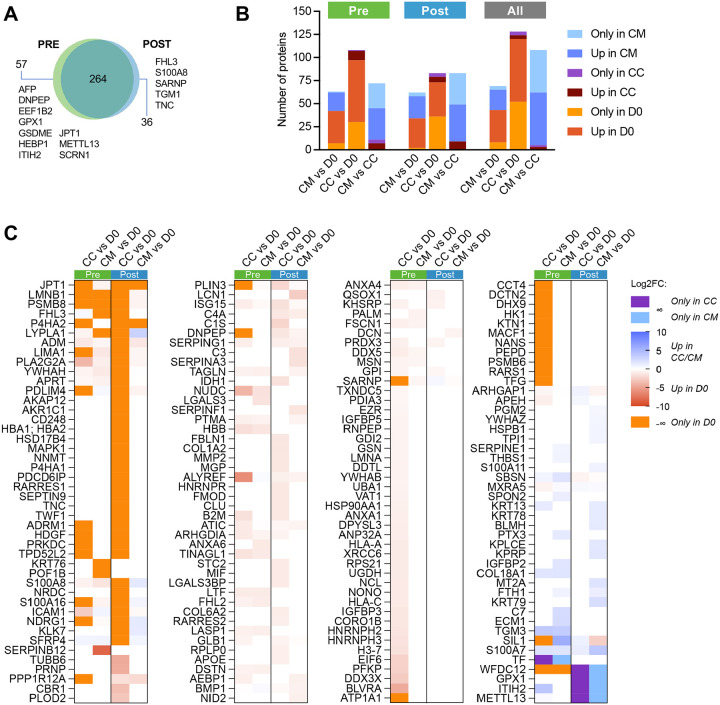
Proteomics analysis of spent media from interstitial cell-derived cultured media (CM) and murine follicle co-culture (CC). (A) Number of proteins detected at baseline (D0) that were present in n≥3 of 5 samples per group. Pre- and Post-specific proteins listed are detected in ≤1 sample of the other group. (B). Pairwise differential expression of proteins between groups. (C) Proteins unique to or differentially expressed in CC and CM vs D0, stratified by Pre and Post samples. For each pairwise comparison (column), proteins present only in one group are colored purple (CC only), light blue (CM only), or orange (D0 only).

Having characterized predicted cell-cell communication patterns from our scRNA-seq data, we sought to identify paracrine factors present in our *in vitro* assay using an unbiased bottom-up proteomics approach. Spent media was collected at the start (day 0; D0) and end (day 7; D7) for all four experimental conditions. When applying a threshold of ≥1 peptide detected in n≥3 samples of at least one group (Pre-D0, Pre-CC, Pre-CM, Post-D0, Post-CC, Post-CM), a total of 501 unique human proteins were identified (**Supplementary Figure 6A-B** and **Supplementary Table 3A-B**). Of these, the majority (n=459 Pre, n=454 Post, and n=484 combined Pre/Post) were detected in all groups (D0, CC, CM) (**Supplementary Table 3C**). To identify the most robust differences in baseline secretome between Pre and Post interstitial cells, we focused on proteins detected in n≥3 of 5 samples in a group; the majority of these (369/445 or 83%) were common between Pre and Post. However, media from Pre-D0 cells included eEF1A lysine and N-terminal methyltransferase (METTL13), alpha-fetoprotein (AFP), glutathione peroxidase 1 (GPX1), and inter-alpha-trypsin inhibitor heavy chain H2 (ITIH2) – none of which were detected in Post-D0 samples (Figure 6A and **Supplementary Table 3D**). GPX1 is known to have antioxidant activity in follicles, and METTL13 may be involved in lipid handling in addition to its role in mRNA translation^64–66^. Alpha-2-macroglobulin (A2M) and aldo-keto reductase family members C1 and C3 were present in all five Post-D0 samples compared to only 2 Pre-D0 samples, while S100A8, which appears to be involved in primordial follicle activation^67^, and tenascin (TNC), an ECM component with increased transcript in ovulatory bovine follicles^68^, were among factors present in 3 Post-D0 samples compared to only 1 Pre-D0 sample (Figure 6A and **Supplementary Table 3D)**.

We next looked at the secretome in the presence of murine follicles (CC and CM). Again, focusing on proteins detected in n≥3 of 5 samples in a group, most were shared between CC and CM (317/417 or 76% for Pre and 324/437 or 74% for Post). ATP binding proteins CCT4, HK1, and RARS1, actin binding protein HDGF, and transport proteins SIL1 and TFG were among proteins detected in Pre-CM but not Pre-CC, while POF1B was found in Pre-CC but not Pre-CM (**Supplementary Figure 6C** and **Supplementary Table 3E**). Proteins in the Pre-CM group corresponded to matrisome-related and protein processing pathways (**Supplementary Figure 6D**). All proteins detected in Post-CC were also in Post-CM in at least one sample, but those present in Post-CM and not Post-CC included A-kinase anchor protein 12 (AKAP12), an FSH-sensitive modulator of protein kinase activity^69^, and KLK7, a secreted protein involved in ECM degradation and proteolysis^70,71^ (**Supplementary Figure 6E** and **Supplementary Table 3E**). Proteins in the Post-CM group corresponded to metabolic processes, including response to steroid hormone (**Supplementary Figure 6F**).

Paired differential analysis was conducted between conditions within each group (CM vs D0, CC vs D0, CM vs CC) (Figure 6B and **Supplementary Table 4A-B**). Due to the high similarity between proteins detected in Pre and Post samples, analysis was also run between conditions for the combined cohort (‘All’) (Figure 6B). First, to understand the effect of interstitial cells on follicle culture, we compared both follicle culture conditions (CC and CM) to baseline (D0) (Figure 6C). Most proteins were unique to or upregulated in D0 compared to CC and/or CM, including growth differentiation factor 9 (GDF9)-regulated collagen synthesis protein prolyl 4-hydroxylase subunit alpha-2 (P4HA2), which has previously been linked to oocyte development and maturation^72^. Few proteins were unique to or upregulated in CC and/or CM compared to D0, but among these was inter-alpha-trypsin inhibitor heavy chain H2 (ITIH2), an ECM stabilizer that was recently found to be upregulated during human oocyte maturation^73^. Notably, some of the proteins in these comparisons overlapped with cell-cell communication modeling from RNA data: COL18A1, predicted to be secreted by SMCs, was upregulated in media from both Pre-CC and Pre-CM compared to Pre-D0, and COL1A2 and COL6A2, predicted to be secreted by theca/stromal cells, was upregulated in Post-CC compared to Post-D0 (Figure 6C).

### Integration of scRNA-seq data with proteomics and immunostaining revealed candidate cell types driving stromal paracrine signaling

**Figure 7.**
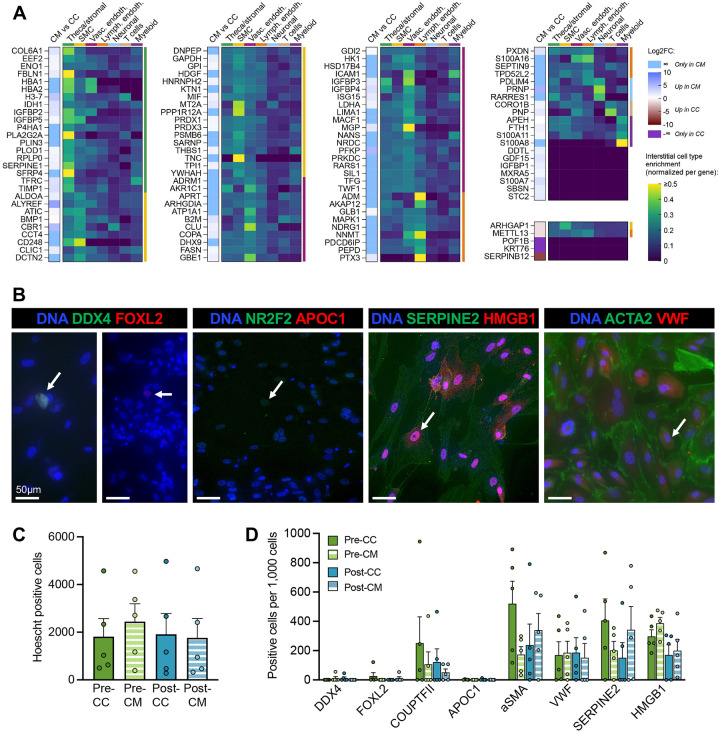
Secreted proteins differentially abundant between co-culture (CC) and conditioned media (CM) experiments and their putative sources. (A) CM- and CC-enriched or unique proteins for all samples (Pre and Post combined) and the average RNA expression of their corresponding genes across interstitial cell populations. Proteins are grouped based on which interstitial cell type is most highly enriched for the corresponding transcript. (B-D) Immunocytochemistry profiling of cultured human ovarian interstitial cells. Representative images (B) show markers for follicular cells (DDX4, oocytes; FOXL2 and SERPINE2, granulosa cells; NR2F2 and APOC1, theca cells) as well as endothelial cells (VWF), smooth muscle cells (ACTA2), and fibroblast activation marker HMGB1. Arrows indicate examples of positive cells. (C-D) Total Hoescht-positive cells per well and (B) cells positive for each marker per 1,000 cells (C) in each experimental condition, mean + SEM. Symbols represent biological replicates.

To determine how follicle-interstitial cell crosstalk affected the secretome, we considered unique or differentially regulated proteins between the CC and CM conditions. With Pre and Post samples combined, there were 103 proteins upregulated or uniquely present in CM and 5 in CC, the majority of which corresponded to transcripts enriched in theca/stromal cells, SMCs, and vascular endothelial cells (Figure 7A and **Supplementary Table 4C**). CM-upregulated proteins were highly enriched for signaling pathways related to apoptosis (e.g., ADRM1, MIF, S100A8), hormone signaling (e.g., ADM, HSD17B4, LIMA1) and ECM organization (ICAM1, P4HA1, TNC) (**Supplementary Figure 7A-D**). Of note, hydroxysteroid 17-beta dehydrogenase 4, which operates in androgen and estrogen metabolism^74^ and whose variants in the corresponding gene *HSD17B4* are causative for premature ovarian insufficiency (POI)^75,76^, was identified in CM but not CC. HSD17B4 is not generally considered a secreted protein; however, its presence in our analysis suggests that interstitial cell-derived HSD17B4 may act as an important signaling molecule facilitating communication between follicles and stroma and may do so through extracellular vesicles. In contrast, premature ovarian failure protein 1B (POF1B), an actin cytoskeleton regulator found in adherens and tight junctions whose gene variants have been causatively linked to POI^77,78^, was present in CC but not CM.

Nearly one quarter (23%) of the proteins uniquely detected in CM were members of gene ontology cellular components extracellular exosome (GO:0070062), secretory granule lumen (GO:0034774), and/or blood microparticle (GO:0072562) (**Supplementary Figure 7E**), indicating a high likelihood that extracellular vesicles are being taken up by cells in co-culture. An additional 19% of proteins were associated with focal adhesions or adhesion junctions. When considering protein-protein interactions, the statistically enriched proteins in CM that were most connected to other identified proteins included POF1B, ICAM1, and S100A8 (**Supplementary Figure 7F**).

To confirm the cells utilized in culture reflected interstitial cell populations like those detected in the scRNA-seq dataset, co-cultured and feeder cells were fixed on day 6 or day 8 of culture when follicles were matured. Interstitial cells evaluated by immunocytochemistry were morphologically heterogenous and expressed markers for multiple cell types (Figure 7B). As expected, follicular cells were rare; on average, only approximately 6 per 1,000 cells were positive for DDX4, an oocyte marker^35,39^, while 10 and 1 per 1,000 were positive for granulosa-specific FOXL2^79,80^ and theca-specific APOC1^35^, respectively. Many more cells expressed markers for generalized ovarian interstitial cells (HMGB1; 26% of all cells), smooth muscle cells (ACTA2; 32% of all cells)^35,80^, and endothelial cells (VWF; 17% of all cells)^35^ (Figure 7C) as well as stromal-theca progenitors (NR2F2 and SERPINE2; 13% and 27% of all cells, respectively)^40,81,82^. Together, these results suggest that the cells secreting factors leading to enhanced growth of murine follicles were representative of native ovarian interstitial populations.

## DISCUSSION

This study utilized a unique cross-species system to define interstitial cell-mediated changes in the ovarian microenvironment across prepubertal and postpubertal periods. Human interstitial cells isolated during routine processing for OTC were leveraged and murine follicles encapsulated in alginate and matured *in vitro* were used as an assay to assess the effects of the prepubertal and postpubertal interstitial cell microenvironment on growth and maturation. There was a robust growth advantage for follicles co-cultured with interstitial cells. Proteomic analysis of media in these cultures identified secreted factors important for follicle growth as well as proteins that may be involved in follicle-stroma crosstalk that could be utilized for *in vitro* experiments to maximize oocyte growth and maturation. In parallel, we performed transcriptomic analysis of ovarian tissue and demonstrated that vascular endothelial cells and SMCs in addition to theca/stromal cells are likely to participate in bidirectional signaling. Overall, these data provide novel characterization of the human ovarian interstitial cell transcriptome and secretome across the pubertal transition, including evidence that paracrine signals promote follicle growth.

Consistent with previous studies evaluating the effect of feeder cell populations on follicle growth^11,12,16,21^, we observed increased follicle diameter in the presence of human interstitial cells. While interstitial cell conditioned media demonstrated a similar growth advantage in the eight-day culture cohort, estradiol levels were only increased when follicles were directly exposed to interstitial cells. This difference in hormone production was not observed when follicles were exposed to interstitial cell-conditioned media alone. Importantly, we did not observe a difference in follicle diameter, survival, estradiol production, or maturation outcomes when prepubertal and postpubertal interstitial cell populations were compared. While individual heterogeneity, cross-species, and/or ovulatory differences may have contributed to these findings, studies across species have documented a relative advantage in egg quality for postpubertal individuals^29,83–89^. These data demonstrate that this growth advantage may be better observed in a system replicating puberty.

To further explore the landscape of cell-cell communication across the pubertal transition, scRNA-seq was performed on tissue from 11 individuals spanning age and developmental stages within the pediatric and adolescent context. Although adult transcriptomic profiles of ovarian cells have been described^32,35,38,40,90^, our dataset fills a critical age and developmental stage gap that highlights transcriptional commonalities and differences of pediatric and adolescent cells compared to the adult. We identified 9 major cell types, including less commonly detected populations of immune cells, neuronal cells, and oocytes. Oocytes and granulosa cells were enriched in prepubertal samples, consistent with previous work documenting increased follicle density in samples derived from a similar cohort^8,91^. The predominant cell type identified within both prepubertal and postpubertal samples was theca/stromal, consistent with protein expression of interstitial cells in follicle co-culture and conditioned media experiments. Analysis of clusters within the theca/stromal cell population revealed 4 subpopulations with gene expression indicative of diversified functions. In particular, the presence of most cells within a single subpopulation, T0, suggests that ovarian interstitial cell populations do not originate as discrete populations and instead exist along a continuum of transcriptomic signatures. This is consistent with lineage tracing studies performed in mice, demonstrating plasticity of interstitial cells to differentiate into follicle specific theca populations^9^. Nonetheless, there was a clear subpopulation, T1, which closely resembled a previously resolved fibroblast subset described by elevated expression of immunomodulatory (e.g., *C3*, *CFD*), cell migration-regulating (e.g., *GSN*, *IGFBP6*) and glucocorticoid- and lipoprotein-related (e.g., *APOD*, *PLA2G2A*) genes^60^. T2 and T3 subpopulations were resolved as theca cells and putative theca progenitors, providing additional evidence for their transcriptional relatedness to other stromal cells.

Considering differences in theca/stromal cells from prepubertal versus postpubertal individuals, we found that genes expressed in prepubertal theca/stromal cells were enriched for pathways including epithelial to mesenchymal transition, response to growth factor, and response to steroid hormone pathways. Genes preferentially expressed in postpubertal theca/stromal cells were enriched for pathways including eukaryotic translation elongation, reactive oxygen species signaling, and P2×7 receptor signaling. Using computational tools to identify putative ligand-receptor interactions, we observed a shift in the source of ligands predicted to signal to theca/stromal cells across puberty. SMC and vascular endothelial cell-derived signals were prominent in prepubertal samples while theca/stromal-derived signals were most prominent in postpubertal samples. This data suggests an increase in autocrine and/or juxtacrine signaling in postpubertal stroma, consistent with bidirectional communication as growing follicles recruit theca cells and become vascularized^19,92,93^.

We next sought to identify secretome signaling networks within our culture system using an unbiased proteomics approach of the spent media and correlating this with scRNA-seq data. Of the 42 significant ligand-receptor pairs in which the ligand was expressed by SMC, vascular endothelial, lymphatic endothelial, and/or theca/stromal cells, nearly one third (14 or 30%) were detected in media of *in vitro* follicle culture experiments, providing evidence that the cultured interstitial cells retained the functional identity of native ovarian interstitial cells. For example, macrophage migration inhibitory factor (MIF) was upregulated in CM compared to CC in postpubertal and combined prepubertal/postpubertal samples but not in prepubertal samples alone. This lends support to the hypothesis that MIF may be involved in ovulation, consistent with a previous study detecting elevated MIF in follicular fluid during ovarian stimulation in IVF and during the ovulatory phase of natural cycles^94^.

Although analysis of scRNA-seq data serves as a useful tool to investigate possible paracine signaling events within the ovary, it is evident that trends do not always correlate to protein abundance and/or utilization. Our proteomics data provides an unprecedented functional readout of human ovarian stromal cells and represents an important foundation for prioritizing mechanistic studies in the future. Several of the proteins significantly enriched in CM (and presumed to be consumed in co-culture) have been previously implicated in ovarian function. Adrenomedullin (ADM), which has been detected in corpus luteum of rat ovaries^95^, may contribute to hormonal signaling between stromal and follicular cells. S100A8 was shown to direct organization of fetal murine FOXL2-expressing stromal cells, presumably pre-granulosa cells^67^. Secreted frizzled related protein 4 (SFRP4) attenuated granulosa cell responsiveness to gonadotropins, thereby decreasing follicle survival, through a GSK3β-AMPK-AKT signaling mechanism^96,97^. Aldo-keto reductase family 1 member C1 and C3 (AKR1C1/AKR1C3), which are associated with extracellular vesicles, are integral to progesterone and prostaglandin conversion, with AKR1C1 localized to luteal cells in the porcine ovary^98^.

This study represents the most comprehensive characterization and functional validation of pediatric and adolescent human ovarian interstitial populations to date – in total, incorporating data from 34 unique human specimens. Efforts to describe these heterogenous and functional populations within the ovary have focused on fetal or adult human ovarian populations, so we aimed to fill this gap in our understanding of human ovarian development across childhood and adolescence. Our results demonstrate the utility of a cross-species assay to answer fundamental questions regarding paracrine mediators of folliculogenesis and provide a functional basis for further exploration of key interstitial-derived signals in ovarian biology.

## MATERIALS AND METHODS

### Ethical approval for the collection and use of human tissue

The collection of human ovarian tissue was approved by the Institutional Review Board of Ann & Robert H. Lurie Children’s Hospital (prepubertal tissue, IRB# 2018-1509; postpubertal tissue, IRB# 2017-1149 and IRB# 2020-3206). Families and participants provided written informed consent for collection of research tissue. When possible, all minor participants provided assent for the use of research tissue.

### Study population

Participant demographics are detailed in **Supplementary Table 1**. Samples were collected from participants undergoing ovarian tissue cryopreservation (OTC) for fertility preservation at Ann & Robert H. Lurie Children’s Hospital (Lurie Children’s) from 2017 to 2023. Determination of puberty status and Tanner stage of participants was performed at the time of consent or within 6 months of unilateral oophorectomy for OTC. Ovaries were removed laparoscopically and transferred to the Northwestern Medicine Andrology lab (before 12/2020) or Lurie Children’s Gonadal Tissue Processing Suite (12/2020 onward) for ovarian tissue processing and cryopreservation performed by certified gonadal tissue processing specialists. At oophorectomy, a 3 mm to 4 mm punch biopsy is taken for routine pathology, where it is evaluated by Lurie Children’s Pathology for the presence of follicles and evidence of malignancy. Tissue found to contain evidence of malignancy was not included in this study.

### Ethical approval for the use of animals

Experiments were performed with 14–16 day post parturition (dpp) CD-1 female mice (Strain 022, Charles River, USA) from an actively maintained breeding colony. All animal work was conducted with the approval of the Institutional Animal Care and Use Committee at Northwestern University (IACUC Protocol IS00015874). Animals had access to drinking water and chow *ad libitum*.

### Ovarian interstitial cell isolation

Ovarian tissue fragments that are released as part of the thinning of the cortical tissue during OTC processes were cryopreserved in Cryostor (#07959, STEMCELL Technologies, Canada) until time of digestion. Tissue was thawed in a 37°C bead bath, washed once in DMEM/F12 (#DFL13-6X50ML, Caisson Labs, USA), and enzymatically digested into a single-cell suspension as previously described^32^. Briefly, tissue fragments were finely minced and incubated in a digestion media containing 40 μg/mL Liberase DH (#05401089001, Sigma-Aldrich, USA), 1000 U DNAse I (#10104159001, Sigma-Aldrich, USA), and 0.4 mg/mL Collagenase IV (#C5138-100mg, Sigma-Aldrich, USA) in alphaMEM + Glutamax (#32-561-037, Fisher Scientific, USA) supplemented with 1X insulin-transferrin-selenium (#25-800-CR, Corning, USA), 1X antibiotic-antimycotic (#ABL02-100ML, Caisson Labs, USA), and 1 mg/mL human serum albumin (#A9511-5G, Sigma-Aldrich, USA). Digestion proceeded for a maximum of 45 minutes at 37°C, 5% CO_2_, shaking at 120 rpm and was quenched by addition of DMEM/F12 supplemented with 10% fetal bovine serum (#F2379-5G, Sigma-Aldrich, USA). Isolated ovarian interstitial cells were seeded into 96-well tissue culture treated plates at a seeding density of 50,000 cells/well. After reaching confluence, cells were treated with mitomycin C (MMC) (#100-1048, STEMCELL Technologies, Canada) to pause proliferation. Mitotically paused cells were allowed to recover in DMEM/F12 supplemented with 10% charcoal stripped fetal bovine serum (#F6765-500ML, Sigma-Aldrich, USA) and 1X antibiotic-antimycotic overnight.

### Murine ovarian follicle isolation, culture, and maturation

Multi-layered secondary follicles were mechanically isolated from 14-16 dpp outbred CD-1 female pups and individually encapsulated and cultured in 0.5% alginate beads using previously described methods^11,99,100^. Only isolated follicles that contained a central oocyte and uncompromised basement membrane surrounding granulosa cells were selected for culture. Culture media consisted of alphaMEM + Glutamax (#32-561-037, Fisher Scientific, USA) supplemented with 30 mg/mL bovine serum albumin (#F2379-5G, Sigma-Aldrich, USA), 1X insulin-transferrin-selenium (#25-800-CR, Corning, USA), 10 mg/mL fetuin (#F3385-1G, Sigma-Aldrich, USA), 1X antibiotic-antimycotic (#ABL02-100ML, Caisson Labs, USA), and 10 mIU/mL of follicle stimulating hormone (EMD Serono, USA). For co-culture experiments, each experiment was divided into three groups consisting of ten follicles per group: co-culture control (alginate encapsulation only, no interstitial cells), Pre-CC (alginate encapsulated follicles co-cultured with prepubertal derived interstitial cells), Post-CC (alginate encapsulated follicles co-cultured with postpubertal derived interstitial cells). For co-culture and conditioned media experiments, each experiment was divided into 6 groups: Ctrl-CC, Pre-CC, and Post-CC as described above as well as Ctrl-CM (alginate encapsulation only, no interstitial cells), Pre-CM (alginate encapsulated follicles cultured in conditioned media derived from prepubertal interstitial cells), and Post-CM (alginate encapsulated follicles cultured in conditioned media derived from postpubertal interstitial cells).

Follicles were monitored over the culture period by brightfield microscopy and evaluated for diameter and survival. Half media changes were performed every other day of culture, beginning at culture day 2. At end of culture (day 6 or day 8), cultured follicles were removed from alginate beads using 10 IU/mL alginate lyase (Sigma-Aldrich, USA) in alpha-MEM (Fisher Scientific, USA), washed in Leibovitz-15 media (Sigma-Aldrich, USA), and incubated individually at 37°C, 5% CO_2_ in 50:50 alpha-MEM and F-12 media (Fisher Scientific, USA) supplemented with 10% charcoal-stripped fetal bovine serum (Sigma-Aldrich, USA), 10 ng/mL epidermal growth factor (Fisher Scientific, USA), 1.5 IU/mL human chorionic gonadotropin (Sigma-Aldrich, USA), and 10 mIU/mL follicle stimulating hormone (EMD Serono, USA) for 14–16 hours. Resultant oocytes were classified as ovulated if the follicle ruptured to release a cumulus oocyte complex and staged by brightfield or immunofluorescence microscopy using established morphologic criteria^101^.

### Follicle co-culture and conditioned media setup and collection of spent media

To generate interstitial cell conditioned media, prepubertal and postpubertal interstitial cells were isolated, plated, and treated with MMC as described above. For day 0 samples, two days prior to co-culture or co-culture/conditioned media experiments, mitotically paused interstitial cells were washed three times in Hank’s Balanced Salt Solution (#MT21023CM, Fisher Scientific, USA) and incubated in 100 μL/well phenol-red free DMEM (#A1896702, Gibco, USA) for 18–24 hours. Spent media was collected and stored at −80°C until lyophilization, and cells were refreshed with follicle culture media prior to follicle isolation and culture for the specified time period as described above. This process was repeated following the end of culture (day 7 samples), and cells were fixed in 3.8% paraformaldehyde (#15710, Electron Microscopy Sciences, USA) + 0.1% Triton-X 100 (#T8787-100ML, Sigma-Aldrich, USA) as described below.

### Sample preparation and digestion for mass spectrometry proteomics

Spent media was lyophilized using a Freezone 6 lyophilizer (Labconco, USA) at Northwestern University’s Analytical bioNanoTechnology Equipment Core. Resultant protein was reconstituted in ammonium bicarbonate (#A6141-500G, Sigma-Aldrich, USA) and in solution digested using mass spectrometry grade trypsin (#PRV5280, Promega, USA) at a concentration of 1 μg trypsin to 10 μg of total protein overnight at 37°C followed by denaturation and disulfide bond cleavage with dithiothreitol (#43816-10mL, Sigma-Aldrich, USA) and iodoacetamide (#786-078, G-Biosciences, USA). Peptide pellets were lyophilized overnight.

### Liquid Chromatography–Tandem Mass Spectrometry with DIA-PASEF

For each sample, 1500 ng of the generated peptides in 0.1% trifluoroacetic acid (TFA) was loaded onto a disposable Evotip C18 trap column (Evosep Biosytems, Denmark) following the manufacturer’s instructions. Briefly, the Evotips were first washed with acetonitrile containing 0.1% (v/v) formic acid, activated by soaking in 2-propanol, and then equilibrated with 0.1% formic acid. The peptide samples were loaded into the tips using centrifugation at 1200 × g. After loading, the Evotips were washed with 0.1% formic acid after which 200 μl of 0.1% formic acid was added to each tip to prevent drying. The peptide-containing Evotips were directly subjected to nanoLC on a Evosep One instrument (Evosep Biosystems, Denmark). Elution was performed onto a PepSep analytical column, dimensions: 150 μm × 25 cm C18 column (PepSep, Denmark) with 1.5 μm particle size (100 Å pores) (Bruker Daltonics, Germany), and a ZDV spray emitter (Bruker Daltonics, Germany). Mobile phases A and B were 0.1% formic acid in water and 0.1% formic acid 80% Acetronitrile (v/v), respectively. Separation was performed using the standard pre-set method of 60 samples-per-day (spd), which is a 21-minute run.

Eluted peptides were analyzed using a hybrid trapped ion mobility spectrometry-quadrupole time-of-flight mass spectrometer (timsTOF HT, Bruker Daltonics, Germany) with a modified nano-electrospray ion source (CaptiveSpray, Bruker Daltonics, Germany). The mass spectrometer was operated in data-independent acquisition PASEF (diaPASEF) mode. Desolvated ions entered the vacuum region through a glass capillary and were deflected into the TIMS tunnel which is electrically separated into two parts (dual TIMS). The first region is operated as an ion accumulation trap that primarily stores all ions entering the mass spectrometer, while the second part performed trapped ion mobility analysis. The dual TIMS analyzer was operated at a fixed duty cycle close to 100% using equal accumulation and ramp times of 85 ms each. For the 60 spd Evosep run, the DIA scheme consisted of one MS scan followed by MS/MS scans taken with 28 precursor windows at width of 25 Th, a cycle time of 0.9 seconds, and a mass range from 300 to 1,200 Dalton. The TIMS scans layer doubly and triply charged peptides over an ion mobility range from 0.7 to 1.3 V*sec*cm^−2^. The collision energy was ramped linearly as a function of ion mobility from 59 eV at 1/K_0_ = 1.4 to 20 eV at 1/K_0_ = 0.6 V sec cm^−2^.

### Proteomics data processing and analysis

Raw files were processed using the DIA-NN software (version 1.9). Spectral library was generated against a combined FASTA file containing sequences for human (SwissProt 2024-09-30), mouse (SwissProt 2024-09-30), and common contaminant proteins. The following settings were applied to generate the spectral library: precursor charge states were set to 2-4, with a maximum of two variable modifications allowed (oxidation of methionine and protein N-acetylation); trypsin/P was selected as the enzyme, with a maximum of one missed cleavage site; protein inference was carried out at the species-specific gene level; mass accuracy for both MS1 and MS2 was set to 13 ppm. Subsequently, any protein groups containing mouse entries were excluded from the DIA-NN report output. The resulting file was then submitted to MSstatsShiny (version 1.6.2), with experimental conditions annotated as described in **Supplementary Table 3A**. The following criteria were applied during processing: only proteins with a Q-value ≤ 0.01 were retained, and proteins with >50% missing values across all runs for a given peptide were excluded. Differential expression analysis was conducted by comparing Group 2 versus Group 1, as detailed in **Supplementary Table 4A**. The results were further processed and visualized using R (version 4.4.2). Proteins were retained for analysis if they were present in at least 3 out of 5 replicates in at least one group. A protein was considered significantly different between conditions if its unadjusted p-value was ≤ 0.05 or if it was uniquely identified in one of the conditions. Significant proteins were further analyzed for Gene Ontology (GO) and pathway enrichment using the clusterProfiler package (version 4.14.4), which included GO Biological Process, GO Molecular Function, KEGG, and Reactome pathways. Pathways were deemed significant if the Benjamini-Hochberg adjusted p-value for enrichment was ≤ 0.05. Protein-protein interaction (PPI) networks were constructed using the igraph package (version 2.1.3), leveraging the BioPlex 3.0 interactomes for 293T and HCT116 cells. Centrality and community structures within the PPI networks were evaluated for the significant proteins.

### 17β-estradiol Enzyme-Linked Immunosorbent Assay (E2 ELISA)

To quantify E2 production throughout culture, spent media was collected from each well during media changes and frozen immediately at −20°C until the ELISA assay was performed according to manufacturer’s instructions (#11-esthu-E01, Alpco, USA). Samples were run in duplicate, and values below the detection cut off for the assay were set to 0.

### Immunocytochemistry (ICC)

Following each experiment, ovarian interstitial cells in all groups were fixed in 1X phosphate buffered saline (PBS) (#SH30258.02, Cytiva, USA) supplemented with 3.8% paraformaldehyde (#15710, Electron Microscopy Sciences, USA) + 0.1% Triton-X 100 (#T8787-100ML, Sigma-Aldrich, USA), washed three times in ICC block [PBS supplemented with 0.1% Tween-20 (#P1379-500ML, Sigma-Aldrich, USA), 0.1% sodium azide (#NC9720916, Fisher Scientific, USA), and 0.3% bovine serum albumin (#A9418-50G, Sigma Aldrich, USA)], and stored at 4°C in ICC block until ICC was performed. Fixed cells were incubated in permeabilization buffer (PBS supplemented with 0.3% bovine serum albumin and 0.1% Triton-X 100) for 15 minutes at room temperature, washed three times with ICC block, and incubated overnight at 4 degrees C in primary antibody at a 1:200 dilution in ICC block (**Supplementary Table 5**). Cells were washed three times in ICC block and incubated in secondary antibody for 1 hour at room temperature in the dark at a 1:500 dilution in ICC block.

Isolated oocytes following *in vitro* maturation were fixed, washed, and stored in ICC block until ICC was performed. Fixed oocytes were incubated in permeabilization buffer for 15 minutes at room temperature, washed three times in ICC block, and incubated in anti-α tubulin antibody conjugated to Alexa Fluor 488 (#8058S, Cell Signaling Technology, USA) for two hours at room temperature or overnight at 4°C in the dark. Nuclei were counterstained with Hoescht 33342 (#B2261-500MG, Sigma Aldrich, USA).

### Single-cell RNA sequencing (scRNAseq) of ovarian tissue biopsies

Ovarian tissue biopsies donated for research during OTC were transferred to the Laronda Lab and cryopreserved via vitrification. When preparing for library prep, biopsies were thawed and digested using previously published protocols^32,102^. Briefly, tissue samples were progressively incubated until equilibration in 0.5 M sucrose (#S9378-500G, Sigma-Aldrich, USA) in basal media consisting of Medium 199 (#M4530-6X500ML, Sigma-Aldrich, USA) supplemented with 21 mM HEPES (#SH30237.01, Cytiva, USA) and 20% dextran serum supplement (9301-100ML, Irvine Scientific, USA). This was followed by 7.5% dimethyl sulfoxide (DMSO) (#D8418-100ML, Sigma-Aldrich, USA) and 7.5% ethylene glycol (#102466-500ML, Sigma-Aldrich, USA) in basal media and finally 15% DMSO, 15% ethylene glycol, and 0.5 M sucrose in basal media. Tissue was either enclosed in the final vitrification solution in a protective straw (IMV technologies, USA) or placed dry in a cryovial (#5000-0020, Thermo Scientific, USA) prior to rapid plunge into liquid nitrogen. Tissue was thawed by first incubating in a water bath set to 37°C, then progressive incubation for 10 minutes per solution in 0.75 M ethylene glycol and 0.25 M sucrose in PBS supplemented with 10 mg/mL human serum albumin (#A9511-5G, Sigma-Aldrich, USA), followed by 0.25 M sucrose in PBS supplemented with 10 mg/mL human serum albumin, and finally PBS supplemented with 10 mg/mL human serum albumin. Following thaw, tissue samples were immediately minced using scissors and digested into a single-cell suspension using the same protocol described above for ovarian interstitial cell isolation. Single cells were assessed for >70% viability at Northwestern University Sequencing Core and immediately processed for scRNA-seq using the Chromium Platform (10x Genomics, USA).

### Single cell RNA-seq analysis

Quality control and cell typing steps were performed as previously described^103,104^. Briefly, gene expression outputs were processed using CellRanger and filtered to exclude doublets and cells with >25% mitochondrial genes using the R package Seurat. Unbiased clustering and uniform manifold approximation and projection (UMAP) was performed using Seurat. Genes specifically expressed by each cluster were examined to identify cell types. Correlation to published cell type signatures was performed using Metascape^105^. To explore the cell-cell communication networks within these gene sets, we analyzed putative secreted ligand-receptor pairs using CellPhoneDB^63^. Functional enrichment analysis was performed using g:Profiler (e112_eg59_p19_25aa4782) with g:SCS multiple testing correction method applying significance threshold of 0.05^106^.

### Statistical analysis & data visualization

Unless otherwise indicated, datasets were analyzed using Prism (version 10.4.1) software (GraphPad, USA) and reported as mean ± standard error of the mean. Statistical significance was determined by unpaired Student’s t-test or 2-way ANOVA using Tukey’s correction for multiple comparisons. A P value of ≤0.05 was considered significant. In addition to Prism, figures were generated using R as well as DeepVenn^107^ and SankeyMATIC (github.com/nowthis/sankeymatic).

## Supplementary Files

This is a list of supplementary files associated with this preprint. Click to download.

- TsuiSupplementaltables.zip
- TsuiSupplementalmaterial.pdf

## Data Availability

Single-cell RNA-seq data will be deposited to GEO, original code will be deposited to Zenodo, and proteomics data will be deposited to PRIDE. All data will be publicly available by the date of publication.
